# Dr. John Forbes on the Use of the Stethoscope and Percussion in the Diagnosis of Diseases of the Chest, with Original Cases and Dissections

**Published:** 1825-01-01

**Authors:** 


*Original Cases with Dissections and Observations illustrating the Use of the Stethoscope and Percussion in the Diagnosis of Diseases of the Chest; also Commentaries on the same Subjects selected and translated from Avenbrugger, Corvisart, Laennec and others.* By John Forbes, M.D. Physician to the Chichester Dispensary. 8vo. pp. 305, three plates. August 1824.

We were the first in this country to give an extended analysis of Laennec's immortal work, and to express our conviction of its superior merits.* We immediately procured some stethoscopes from Paris, and had others made in London, whence a very great number were diffused among the profession. We much doubt, with Dr. Forbes, that the discovery of mediate auscultation has not been appreciated, as it deserves, in these kingdoms—at least, if we may judge from the paucity of notices respecting it in the public journals.

Dr. Forbes, therefore, deserves great praise, not only as the translator of Laennec's work, but as an able cultivator and zealous promulgator of the science of diagnosis as applied to diseases of the chest. By careless routinists, and even by many from whom we might expect better things, minute diagnosis is despised, because, say they, the same treatment applies to the greater number of those diseases that are likely to be confounded—while in those that are incurable it is useless, as all treatment is unavailing. It is certainly true that precise discrimination of different diseases or different shades of the same disease, may not at present, or perhaps ever, increase our power of curing or even relieving them. But it may prevent our doing harm:—it may prevent our pursuing means that are useless or injurious—and it *will undoubtedly* enable us to avoid a false diagnosis and prognosis on very many occasions, and give a true one, by which our reputation becomes enhanced, and the dignity of the science maintained. When insisting on the advantages to be derived from auscultation and percussion, we are very far from believing that these means are to supersede in the slightest degree the common means generally pursued—namely, a minute inquiry into the history of the disease and mode of life of the patient, together with a very particular examination of the various vital and natural functions. Very often when we cannot cure or make any impression on the main or ultimately fatal disease, we can yet so ameliorate certain symptoms consequent on it, and relieve certain functions disturbed sympathetically by it, as to render the remainder of life comparatively comfortable. We dwell the more earnestly on the common means of investigating diseases, because, in this country at least, we greatly fear that auscultation, particularly by means of the stethoscope, never can come into general use *in private practice*. In hospitals and other institutions of that kind, however, the physician has opportunities of cultivating this mean of diagnosis, and he is culpable not to do so, because *occasionally* he will be able to turn his knowledge to account

---

* Medico-Chirurgical Journal, January 1820, pp. 461, et seq.

in the private walks of his profession. We shall give an illustration from Dr. Forbes' preface.

"Suppose two physicians of equal skill and experience, but only one an Auscultator, met in consultation, for the first time, on a case of Chronic Pleurisy or Peripneumony, in no very advanced stage. Probably the only remarkable symptoms are, a violent cough with little or no expectoration, more or less dyspnœa, especially on motion, and incapacity to assume, with comfort, certain postures; the pulse perhaps is natural, there is no pain, no febrile heat, no hectic, and inconsiderable loss of flesh; or, perhaps, one or other of these states may exist. From these general symptoms, will he who is not the Auscultator venture to pronounce, with any degree of confidence, his opinion of the precise nature of the disease; much more, to declare it to be one that will almost certainly prove fatal, and scarcely even admit of relief from any kind of treatment? No practitioner, I affirm, judging from the common symptoms only, can be justified in entertaining such opinions. But if, to the examination of the symptoms, be superadded the exercise of percussion, or the exploration by the stethoscope, for only two or three minutes; and if the former elicits the preternatural or fleshy sound over the whole of one side of the chest, or the latter indicates the absence of respiration over the same space; then, at once, are obtained sufficient materials (conjointly with the previous history and present symptoms of the case) to form a correct diagnosis, and to justify and authorise the most ominous prognosis. Were the benefits of Auscultation confined to this single case of disease, it would, even then, be entitled to the highest consideration." xviii.

Our author makes some very judicious reflections on the error of over-rating pathology, at least *post-mortem* researches. We have often called the attention of our readers to this subject. On the Continent they do not look sufficiently to the preceding functional disturbances that lead to organic changes of structure. All their attention is fixed on what remains to be seen after death. Our author well observes that—"this mode of judging of the character of diseases is scarcely more just than would be that of the biographer, who would draw the character of his hero from the last act of his life only, without any regard to his previous conduct." Dr. Forbes begins, like most men who are conversant with disease, to be sceptical as to the power of medicine over changes of structure. Nature no doubt, sometimes removes organic changes by the same powers with which she has produced them—and she may be stimulated to this operation by medicinal agents. But undoubtedly we can seldom calculate on such fortunate occurrences. Our main hopes rest on correcting or altering *morbid function*. Our Continental brethren are too inert in their measures for this purpose—and many, very many of the British faculty are too active in the ulterior stages of disease, where morbid function has ended in, or rather produced, disorganization. In this way our author thinks, that "the accounts between the two countries may come, in the end, to be nearly balanced." In this judgment we can hardly agree with our excellent author. We hold it to be an axiom that life is more endangered—that the sum total of mortality is more augmented by inert measures in the beginning, than by too strong measures towards the close of an acute disease. The Hippocratic practice suffers curable to pass into incurable disorder—heroic remedies and ultra-depletion, in late stages of disease, where structure has become changed, only precipitate a fate that would be pretty certain, whatever was the practice. In this respect the British must be considered superior to the Continental methodus medendi. Indeed it is quite evident that the French themselves are opening their eyes to the dangers of the *medecine expectante*, for scarcely a month passes without seeing some reflection thrown out against it, and some advance to a more energetic manner of treating acute diseases.

"The practical conclusion from all this ought to be, not to disregard pathology, but only to cultivate it in a more philosophic spirit. To derive benefit from our dissections, we ought quite as much to reflect on what was the cause and early character of the organic change which we see consummated, as to study the actual nature and final amount of this change. Without this retrospective ratiocination, and practical dedication of his researches, the morbid anatomist is more an artist than a physician; and his devotion to science is, to use the language of Bacon, rather idolatry than true worship." xxii.

While it is the object of the volume before us to recommend auscultation and percussion, he warns the profession against an exaggerated expectation of its applicability. He thinks, and with reason, that in the greater number of the more recent inflammatory affections, auscultation may be dispensed with, the common symptoms being generally sufficient to regulate our practice. At the same time he appears to think that, even here, percussion and the stethoscope would render our judgment and practice, in many cases, more satisfactory and certain. It is in *chronic* diseases, of course, that these diagnostic means are of the greatest importance.

We beg leave to suggest the propriety of simple or immediate auscultation with the ear, as little inferior to that with the stethoscope, and much more applicable to private practice. In examining the chests or abdomens of patients affected with chronic diseases, we invariably strip them so as to see the conformations of the thorax and abdomen, as well as to use thoracic percussion and abdominal compression. We then find no difficulty in adding, if we see occasion, auscultation with the naked ear. But certainly the application of the stethoscope carries with it an *appearance* of quackery, which it is desirable to avoid, at least among the higher ranks of society, who are apt to canvass a medical man's actions, as well as words. In hospital practice, as we before observed, the stethoscope should be diligently studied and applied.

But we must hasten to a conclusion of Dr. Forbes's preface. He asserts, and we are convinced that his assertion is most true, that in publishing this volume he is actuated by the very best motive that a medical man can alledge—namely, the conviction that the work is wanted and that it will prove useful to his brethren.

As it now stands, the publication consists of, *first*, a translation of Avenbrugger on percussion, with the valuable commentaries of Corvisart—*Secondly*, a brief view of the principles and practice of mediate auscultation, reprinted, with inconsiderable alterations, from Dr. Forbes's translation of Laennec, now out of print—*Thirdly*, a series of *original cases* in illustration of auscultation, percussion, &c. and *lastly*, an appendix containing a compendium of Dr. Collin's Essay on the different modes of exploring the chest.

The work of Avenbrugger was published more than sixty years since. Although it is acknowledged to contain false theories and incorrect opinions, it is certain that it also contains many sterling truths, the discovery or perfection of which has been attributed to more modern writers. The author was born in Styria in 1722, and, it is said, he lived till 1809. Little is known of him, except that he wrote also on madness, and on dysentery. He is reported to be the author of a *drama*. The work on percussion made little impression in Germany, and was still less known practically in France, till the year 1788, when Corvisart introduced it, and enforced it by precept and example. After twenty years' experience, this celebrated physician established the practice on a lasting foundation by his translation of, and admirable commentaries on, the original treatise of Avenbrugger. The practice has long been extensively employed in France, and especially in Paris, as a common and indispensable measure in studying diseases of the chest. How little it has been attended to in this country may be inferred from the circumstance that it is not even named in the best popular elementary or systematic works—E. G. in the publications of Parr, Gregory, or Good!—It is very certain, however, that the late, lamented Dr. Baillie employed percussion pretty generally in the investigation of thoracic diseases—and that probably no inconsiderable share of his reputation was owing to this practice as an auxiliary to correct diagnosis and prognosis. Examination of the chest by percussion, and of the abdominal viscera by pressure, is now practised by several of the best pathological physicians in this metropolis—and no doubt in various parts of the kingdom. The principles on which the practice is founded are so very little known, that we shall dedicate the greater part of this article to a pretty full enunciation of them, as contained in Avenbrugger's work and Corvisart's commentaries.

### PERCUSSION OF THE CHEST.

"*La percussion de la poitrine est, sans contredit, l'une des découvertes les plus precieuses dont la medecine se soit jamais enrichie.*"—*Laennec.*

We shall not particularly adhere to the distinctions between the text of Avenbrugger—the commentary of Corvisart—or the notes of Dr. Forbes—our object being to convey a general idea of the whole subject, as collected from all.

The sound elicited by percussion from a healthy chest resembles the stifled sound of a drum covered with a thick woollen cloth or other envelope. The method of eliciting this sound, in other words, of applying percussion, is first to be described.

The thorax ought to be struck gently with the points of the fingers brought close together, but at the same time extended. Percussion with the flat open hand is also sometimes useful, especially on the lateral and posterior parts, (so thinks Corvisart) and therefore the two modes may sometimes be conjoined in the same case. In fat and robust subjects the percussion should be stronger; and in all cases we are to apportion the impulse according to the thickness and other circumstances of the parietes, for in this consists the great art of *applying* percussion. If it is not agreeable to the patient to be entirely stripped, the shirt or chemise should be drawn tight over the part to which percussion is applied. The sitting posture is the best for the patient, who should be directed to hold his head erect, and keep the shoulders back, in order that the chest may protrude, and the skin and muscles be drawn tight over it.—This is, of course, when the fore part of the chest is to be struck. During inspiration and the retention of the breath, the sound is every where louder than during expiration. While striking the ribs, or lateral parts of the chest, the patient should be directed to hold his arms across his head, for the obvious reason of rendering the thoracic parietes more tense. In operating on the back, the person is to bend forward, and draw his shoulders towards the anterior part of the chest, for similar reasons. It is obvious that in some acute diseases we cannot secure these advantageous positions; but we ought to come as near to the above rules as possible. Nothing but exercise of percussion on sound and unhealthy subjects can give us the *tactus eruditus* in this mode of exploring the chest, and distinguishing its diseases.

Having laid down the mode of applying percussion, let us now see what is the result.

The sound, previously described and which may be made familiar to the ear by trying on healthy subjects, is perceptible in different parts of the chest, as follows:—On the *right* side *anteriorly*, it is observed from the clavicle to the sixth true rib —*laterally*, from the axilla to the seventh rib—and *posteriorly*, from the scapula to the second and third false ribs. Disease of the liver, however, or other abdominal organs, by encroaching on the chest, will sometimes contract the aforesaid sonorous sphere. On the *left* side, the sound can generally be heard lower down than on the right, owing to the absence of the liver, and the presence of the stomach or colon containing air. The sound obtained by percussion over the region of the heart varies extremely in different persons. In those that are spare, with a weak pulse, or where the heart is small, the sound is scarcely at all altered in the region of that organ—while in others, of opposite temperament and habit, it is with difficulty perceived.—Disease will sometimes produce the same effect. Thus in phthisis, with much emaciation, the presence of the heart seems hardly to weaken the sound at all. The whole sternum yields as distinct a sound as the sides of the chest, except in the cardiac region, where it is somewhat duller, though this difference is seldom perceptible. That part of the spinal column which contributes to form the chest emits a sound, but duller than the sides of the thorax.

In fat or very robust persons, the sound is very dull or almost lost. The most sonorous region is from the clavicle to the fourth rib anteriorly. Lower down, the mammæ and pectoral muscles deaden the sound.

So much for the natural sound in health. We now necessarily come to the preternatural or morbid sound, emitted by the chest during percussion—and its general import. It is almost needless to observe, that the healthy sound and all its modifications, according to situation or habit of body, should be first studied, before we can attempt to appreciate its deviations in disease, in the same way that common anatomy must be learnt before we can judge of morbid changes of structure.

1. If then a distinct sound, equal on both sides, and commensurate to the degree of percussion, be not obtained from the sonorous regions above-mentioned, a morbid condition of some of the parts within the chest is indicated. "The least sonorous side (Corvisart) is certainly diseased." Avenbrugger and Corvisart aver that "diseases of the worst description may exist within the chest, unmarked by any symptoms, and undiscoverable by any other means than percussion alone." A clear and equal sound elicited from both sides of the chest indicates that the air cells of the lungs are free, and uncompressed by either a solid or liquid body, with exceptions which will be mentioned anon. Where the sound then is good, the practitioner may feel assured, that, however much the chest may seem to be engaged, there is, as yet, no organic lesion of consequence there. This conviction will allow his observation to be directed to other parts of the body, where may be the real seat of the affection, some of the sympathetic effects only of which had previously engaged his attention.

If a sonorous region of the chest be entirely destitute of the natural sound, on percussion, yielding only a sound like that of *a fleshy limb*—in that region disease exists, commensurate with the extent of unnatural sound. If this bad sound continues when the patient holds in his breath after a deep inspiration, disease extends profoundly into the cavity of the chest. If the same results are obtained both before and behind, on points precisely opposite, we may conclude that the disease occupies the whole diameter of the chest. In short, under such circumstances, we are authorized to conclude that the obstructing body either entirely pervades, or has, in a great measure, displaced the lung, and excluded the air. "Such varying results," says Avenbrugger, "depend on the greater or less diminution of the volume of air usually contained in the thorax (lungs); and the cause which occasions this diminution, whether solid or liquid, produces analogous results to those obtained by striking a cask, for example, in different degrees of emptiness or fulness: the diminution of sound being proportioned to the diminution of the volume of air contained in it." This illustration is no less happy than just—but it affords us no criterion whether the obstructing body be solid or fluid. Such criterion Corvisart avers that he has discovered by placing the patient, while under percussion, in different positions, as in the erect and horizontal. If the obstruction consist of a *fluid*, it will vary its level, with a corresponding variation of sound—on the other hand, if it be a solid, the same results will be obtained in every position. This, of course, will not hold good when the cavity of the chest is quite full of fluid, or where there is chronic pleurisy occupying only a part of the chest, in which case, the fluid is generally confined by false membranes. It is only applicable to simple hydrothorax.

2. The application of these principles to diseases.

It may be stated as a general fact, according to Corvisart, that all diseases of the chest, whether acute or chronic, occasion the preternatural sound, either during their progress, or towards their termination. He does not even exclude those nervous asthmas and violent coughs which appear, in the first instance, at least, to depend on irritation of the nerves of the part. In almost all these cases, we find, after death, some induration, effusion, or other morbid change.

"In peripneumony," (says Corvisart) "and pleuro-peripneumony, if the inflammation is acute, the morbid sound is perceptible on the second, third, or fourth day at farthest: the natural resonance gradually lessens, and is entirely lost by the sixth or seventh day, if the disease is to terminate fatally. On the other hand, it gradually reappears if a recovery takes place. In the more severe and simpler cases, the common symptoms are less equivocal; but in the more complicated and obscure examples of the same disease,—without percussion, how shall we ascertain the real state of the lungs, or be able to avoid committing ourselves by unwarranted opinions and false prognostics? In such cases, effusions into the pleura, or vomicæ take place insensibly; and frequently under the deceitful mask of an amendment of symptoms, or even of convalescence. The result, however, soon shows the false security of the practitioner. These grievous mistakes can only be avoided by the employment of percussion." 19.

The following corollaries are drawn by Avenbrugger, as the result of his observations in inflammatory diseases of the chest, studied under the sign of morbid resonance.

1. The duller the sound, and the more nearly approaching that of a fleshy limb stricken, the more severe is the disease.—2. The more extensive the space whence morbid sound proceeds, the more certain is the danger from the disease.—3. The disease is more dangerous on the left than on the right side—probably from the vicinity of the heart.—4. A morbid sound emitted from the superior and anterior part of the chest (from the clavicle to the fourth rib) indicates less danger than from the inferior parts of the thorax.—5. The want of natural sound behind indicates more danger than on the anterior and superior parts of the chest. This is sufficiently accounted for by the greater volume of the lungs behind.

"6. The total destitution of sound over one whole side, is generally (passim) a fatal sign.

"*Comm.* This total want of the natural sound over one whole side of the chest, may arise either from a hepatization of the lung, or from an effusion into the cavity of the pleura: in either case, I am of opinion with *Stoll* (Aph. 140), that, if not always, at least in an immense majority of cases, the event is fatal.

"¶. My own experience," says Dr. Forbes, "and (what is of much greater authority with me) the testimony of Laennec, would lead me, on the contrary, to believe, that the proportion of fatal cases, under such a sign, is not so immense. In that particular termination of acute pleurisy, so beautifully described by Laennec under the name of 'contraction of the chest'—and of which instances are by no means extremely rare,—the natural sound is not only wanting over the whole affected side, but, according to the author just mentioned, never returns, even after the recovery of the patient." TRANS. 23.

7. The absence of sound along the sternum is a fatal sign. On this Corvisart observes, that, "a collection of matter in the anterior mediastinum (the only case likely to produce this sign) is generally, though not always a fatal affection."—8. The entire absence of the natural sound over a large space in the region of the heart, is a fatal sign. This, Corvisart thinks, indicates an inflammation, acute or chronic, of the pericardium (or perhaps the heart) with a purulent or serous effusion in this cavity.

3. *Chronic Diseases.* The preternatural sound emitted in chronic diseases is owing, according to Avenbrugger, either, *first*, to some hidden condition of the organs, disordering, and finally destroying them by a slow process—or, *secondly*, exists when certain obvious causes have induced a slow disorganization of the same. Percussion is therefore proper in all chronic diseases—for whether we know the precise cause or nature of the disease, it will at least be an advantage to know that there *is* organic disease. Under the first class of causes Avenbrugger ranges hereditary predisposition—affections of the mind, particularly nostalgia—and certain trades or professions. Nostalgia seems to have occupied a good deal of our author's attention, and he avers that it is a strong cause of pectoral disease. "I have examined," says he, "the bodies of many youths who have fallen victims to it, and have uniformly found the lungs firmly united to the pleura, and the lobes on that side where the obscure sound had existed, callous, indurated, and more or less purulent." In respect to trades, he has particularly remarked that tailors, millers, &c. who are forced to inhale, during their labours, a fine dust, become phthisical—while shoe-makers, weavers, &c. from the forced position or application of their weak chests, become asthmatical, with indurated lungs, &c. The following is Corvisart's commentary on nostalgic affections.

"All the great and more profound mental affections are powerful causes in the production of corporeal disease. They principally affect the functions of the organic life: the organs concerned in circulation, respiration and digestion are especially modified; but the functions of secretion, exhalation, absorption, and nutrition, which seem at first untouched, eventually undergo no less essential changes. These organic modifications vary with the particular causes. Thus anger, by directly acting on the circulation, occasions dilatations of the heart, &c. Joy, on the other hand, merely augments the actions of the same organs in a slight degree, and determines the blood toward the surface. Grief produces a directly opposite effect: it debilitates the whole vascular system, obstructs the course of blood towards the capillaries, and thus occasions that general paleness, which is most conspicuous in the face: from this result a deficiency of blood on the surface of the body, and a superabundance in the larger vessels,—occasioning the sighing, oppression, and palpitations, the passive dilatations of the heart, the engorgement of the lungs, their adhesion to the pleura, and the diminished resonance described by Avenbrugger. To these causes may be added the derangement of digestion, which is found to be an inseparable attendant of long-continued moral affections, and which inevitably induces such a state of general debility as greatly predisposes our various organs to be acted on by the morbific causes to which they are exposed.

"It is to be regretted that Avenbrugger confined his anatomical examination of such cases to the lungs; as I am led to believe from the causes and symptoms of the disease, that the heart and great vessels would have been found equally affected." 27.

Here both Avenbrugger and Corvisart give testimony to the insidious nature of pulmonic affections, which simulate various diseases or lie masked themselves, thus completely deceiving both patient and physician.

4. *Extravasations within the Chest.* When, from any cause, there is an effusion of fluid, in any considerable quantity, the preternatural sound will exist over the space occupied by it.

5. Our author properly observes, that there are many apparent diseases of the lungs, which have their real seat in other parts, especially in the abdomen. These affections rarely give rise to the preternatural sound. "From the absence of this, and the presence of a copious watery urine, their existence (as sympathetic affections) may be pretty confidently presumed." We shall give Corvisart's commentary on this point, as it exhibits a considerable coincidence with the writings of Dr. W. Philip and others in this country.

"Most of the affections mentioned in the preceding paragraph, I consider as purely sympathetic, and as depending, in general, on disorder of the digestive organs. I agree with the author that such affections are, in their earlier stage, not discoverable by means of percussion; but as nothing is more common than the conversion of those disorders which are called nervous, into affections truly organic of the heart or lungs, there is no doubt that eventually they become discoverable by this means. The origin of pulmonary disorders, in nervous affections attended by a cough is easily explained. Besides, who has not observed that a great proportion of nervous women die phthisical? It is also true that disorders of the stomach induce pulmonary phthisis. In long continued derangements of the digestive organs, congestions will almost certainly ensue in some of the abdominal viscera, which while they impede the function of respiration (mechanically or by sympathy), will necessarily in the end affect the lungs themselves. The continued irritation will produce an indolent state of phlogosis, which may be considered as the first stage of those structural lesions which constitute phthisis; and which will be followed by tubercles, &c. I may add, that those secondary consumptions, whether the consequence of nervous affections or gastric disorder, would be of extremely difficult recognition, but for the aid afforded by percussion,—inasmuch as it is their peculiar character, that the symptoms of the primary disease still continue, for some time, the more conspicuous of the two." 31.

A slight engorgement of the lung, an hepatization to a trifling extent, a small vomica, an inconsiderable extravasation, are not detected by percussion. Corvisart, however, seems to think that an experienced hand would detect a vomica the size of a common walnut, if situated on the surface of the lungs.

6. *Appearances on Dissection, where morbid sound has existed.*—These are scirrhus (he means what we now denominate induration or hepatization)—vomica—purulent vomica—empyema—dropsy—extensive extravasation of blood—aneurism of the heart. Corvisart's commentary on hepatization, is as follows:—

"I am ignorant of the precise nature of this affection. I think it is generally formed slowly and insensibly, as in the case of artisans exposed to a dusty atmosphere, or as a sequel of acute disease. Perhaps, also, it may originate spontaneously, and be the result of a very slow latent peripneumony, &c. The term *carnification*, lately applied to this affection, is inexact, inasmuch as the character of the lung is not at all like muscular *flesh*, but like *liver*: on this last account the term *hepatization* is much more correct and appropriate. In this variety of morbid structure, what becomes of the blood-vessels, bronchia, vesicles, glands, and nerves of the pulmonary organ?" 34.

As this hepatization is a very frequent occurrence, and very difficult of detection, by symptoms alone, we shall quote the observations of Avenbrugger on this point.

"Together with the diminution or entire loss of the natural sound over the affected part, there is an infrequent cough without any expectoration, or with only a scanty excretion of viscid and crude sputa.—During a state of quiescence there is nothing to be observed much amiss, either in the condition of the pulse or respiration; but upon any considerable bodily motion, or after speaking for some time, these persons become speedily exhausted, anxious, and breathless, and complain of a sense of dryness and roughness in the throat. At the same time the pulse, which had previously been of moderate frequency, becomes quick and unequal; the respiration and speech are broken and interrupted by sighs; the temporal, sublingual, and jugular veins of the affected side, are more than usually distended; while it will be observed that this side of the chest is less moveable than the other, during inspiration. Meanwhile the natural and animal functions continue to be well performed; and the patient can lie on either side indifferently." 36.

To the foregoing signs of pulmonary hepatization, which are exceedingly just, Corvisart has added the following, which are also very valuable. He avers that they are *invariably* present in hepatization.

"1. *Progressive emaciation,* although sufficient nutriment be taken;—2. a low febrile diathesis;—3. a feeling of oppression and weight, but without pain, in the affected side. From the manner in which the author terminates his description of this disease, one might be led to imagine that it might remain stationary without necessarily abridging life. The truth however is, that it proves fatal more frequently in its indurated, than in its suppurated state. When it has reached the height indicated by the signs above detailed, it increases rapidly, and its fatal termination is precipitated by additional symptoms. The appetite gradually decreases; the sleep is lost; great anxiety prevails; and the character and temper are entirely changed, the patient (although naturally placid) often becoming harsh, violent and irritable, and extremely desponding. There are partial sweats on the breast and neck, sometimes warm, more frequently cold, and followed by fainting. There ensue frequent paroxysms of extreme breathlessness. The patients are now confined to bed in a state of complete marasmus. Some can lie on the back; others can bear only a sitting posture; in the last days of life none can lie down except on the affected side. Sometimes there is slight delirium, sometimes not; sometimes there is œdema of the limbs of the affected side, but more commonly there is none. Before death the pulse becomes small, unequal and intermittent." 37.

In respect to the *progressive emaciation*, we can vouch, from our own observation, that this symptom is not *invariably* present in hepatization of the lungs. Within these few months, two instances occurred in our own practice which shake the foundation of Corvisart's rule. One was a young lady, whom we examined in presence of Dr. Copland, who had been epileptic from birth, but who died rather suddenly, bringing up purulent matter. One lung was completely hepatized, except where some vomicæ, situated in it, were broken down, and communicating with the principal ramification of bronchia in that side. Yet this young lady was very far from being in a state of emaciation, although harrassed with cough for some years. We were curious in this instance to examine the brain of a person epileptic from birth till the 34th year, when she died. We found the skull excessively hard—and the substance of the brain itself was of such a dense consistence, in several places, that it cut like leather. The basis cranii was exceedingly jagged with sharp spiculæ of bone, and all the ridges and prominences were remarkably acute and pointed. The other case was that of a lady in Chandos street, who also died suddenly when apparently convalescing from pleuritic inflammation. The pain of side, the difficulty of breathing, the cough, and the pyrexial heat had subsided, under the usual means; but the pulse kept up above 120, with a cool skin, face rather pale, and some tendency to delirium. She was suddenly seized with a sense of suffocation in the night, and expired after a few hours' struggle, without bringing up any expectoration. We found three-fourths of the right lung completely hepatized, so that no trace of its natural organization remained visible. There were some tubercles in the left lung, and an effusion of muco-purulent matter into the bronchia. In this case there was disease of the heart, the parietes of which were remarkably extenuated, and so flabby that the finger easily went through the substance even of the left ventricle. Dr. Henry Davis, Mr. Morris, and Mr. Wray, were present during the examination. This lady had been subject to cough, dyspnœa, and pain in the right side for some years; but she was so uncommonly embonpoint that the fat on the sternum was more than an inch in thickness. We could adduce other instances of a similar kind; but the above are sufficient to shew that exceptions to Corvisart's rule are not of very rare occurrence.

Avenbrugger's remarks and Corvisart's commentaries on the subjects of vomica and empyema are not particularly interesting, in the present state of our knowledge on these points. We shall therefore pass them over.

*Hydrothorax.* Avenbrugger was not careful enough to distinguish idiopathic from symptomatic hydrothorax. The *former* disease is doubtless very rare, as Corvisart has justly remarked —the latter very common, in consequence of the great number of organic diseases by which we are afflicted, and of which hydrothorax is only a symptom or effect. The commentary of such a man as Corvisart, on Avenbrugger's list of symptoms attending hydrothorax, is worthy of a place in this journal.

"*Comment.* The author's distinction of hydrothorax according as one or both sides are affected, although useful and proper, and easily known by means of percussion, is merely a difference of *quantity*, and would, I think, be better expressed by the terms *partial* and *complete*. But the essential grounds for different species of dropsy is a difference in their nature and causes; and hence the grand natural division of these must be into *Primary* and *Symptomatic*. Without this distinction there will always be the greatest confusion in characterising them by any signs or symptoms.

"[I shall now remark, in order, on the various symptoms enumerated by the author, and shall repeat the notation and titles employed in the text, for the sake of easy reference.]

"1. *Difficult respiration.* The respiration, strictly speaking, is not difficult or laborious, except on exertion; it is short and confined, yet performed with sufficient calmness. The cause of the short breathing is obvious.—But even if the respiration were laborious, this would still be but a very equivocal symptom, as it exists in many other affections, e. g. in all the diseases of the heart, large vessels, or lungs; in true asthma; &c.

"2. *Cough, &c.* The intervals between the fits of coughing are long, and the cough itself is generally inconsiderable and without any violent paroxysms.

"3. *The pulse.* The characters of the pulse in the idiopathic hydrothorax, are absolutely the reverse of those given by our author,—it being, most commonly, *full, somewhat soft, slow, quiet, regular*,—feebler and more frequent according as the complaint increases, but always remarkable for regularity. These, at least, are the qualities of pulse which I have observed in similar cases, and I have had occasion to witness a great many, considering the infrequency of this disease in its state of simplicity.

"In organic diseases of the heart and great vessels, however, one of the principal symptoms is the deranged condition of the circulation,—the pulse being, at different times, *hard, full, vibrating, frequent, irregular, greatly intermitting, undulating*, in short, varying extremely. It would appear, then, from this statement, that in the cases of hydrothorax, wherein Avenbrugger had observed the kind of pulse mentioned by him, —there existed some primary organic lesion of the heart or large vessels, of which the hydrothorax was merely a consequence;—as it is known to be one of the most common.

"4. *Breathlessness on motion.* This symptom is always present, and is easily explained on mechanical principles. It is, however, a very equivocal sign of hydrothorax, since it is found in all other diseases which produce a proportionate impediment to the introduction of the necessary quantity of air into the lungs,—such, for instance, as a scirrhus or vomica of the lungs, empyema, aneurism, &c.

"5. *Nausea, &c.* I consider this sign as insignificant.

"6. *Præcordial anxiety.* This symptom is not usually present in idiopathic hydrothorax, but commonly attends the cases resulting from disease of the heart. It is, therefore, an equivocal mark of simple hydrothorax.

"7. *Oppression of the chest after eating.* This is readily explained by the pressure of the enlarged stomach on the diaphragm.

"¶ I wonder that the commentator does not remark the equivocal character of this symptom also, since it exists in almost all diseases of the heart and pericardium. TRANS.

"8. *Flatulence.* The discharge of this gives momentary relief in hydrothorax, on the same principle that the retention of it occasions uneasiness: it cannot, however, be properly reckoned as a symptom of this disease in particular.

"9. *Thirst.* I consider (with the author) this to be very rare in the idiopathic affection, but not so in the symptomatic.

"10. *The Urine.* The state of this excretion described by the author is common to every species of dropsy.

"11. *Epigastric tumour.* Explained on mechanical principles.

"12. *Livid œdema of the extremities.* This symptom rarely exists in primary hydrothorax, and is rather to be considered as a sign of organic disease of the heart.

"13. *Œdema of the eyelids.* This symptom is certainly common in primary hydrothorax, but exists also in many other affections.

"14. *Paleness or Lividity of Countenance.* In hydrothorax the face is generally pale, wasted, anxious, and not swollen; the lips pale and thin; the tongue neither pale nor livid. All the symptoms of lividity depend on an affection of the heart or great vessels.

"15. *Anxiety, inability to lie down, &c.* I would say that none of the symptoms here enumerated belong to simple hydrothorax, which is a disease of comparative tranquillity and sameness. In the same affection, however, resulting from diseased heart, all the symptoms enumerated exist in a high degree." 51.

The veteran Corvisart makes a remark here, the justice of which we have had several opportunities of witnessing; namely, that in hydrothorax, even of both sides of the chest, the patient is often able to lie down in the horizontal position. He avers, however, that this ability is never seen in hydrothorax dependant on disease of the heart. Certainly in those cases where we had opportunities of dissection, and where this unexpected ability had obtained, there was not disease of the heart, though generally of some other organ. Still we are disposed to think Corvisart's rule too positive, as indeed are most of his rules. Corvisart is doubtless right when he observes that simple idiopathic hydrothorax is a very rare disease. His description therefore of such a malady is valuable, since few have had such means of laying down their diagnostic didactics on the extensive basis of *post-mortem* examination. We shall give his description, as it is very concise.

"In recognising this affection, it is important to study its causes and the previous history of the individual. When idiopathic, its attack is generally sudden, and succeeds to cold drinks—the suppression of accustomed evacuations,—the measles, &c. &c. When any of these causes have preceded the disease, and none of the symptoms peculiar to organic lesions of the lungs, heart, or great vessels have done so,—there exists already much probability of the uncomplicated nature of the dropsy.

"The patient at first complains of a sense of weight, uneasiness and weakness, of anxiety and pressure at the præcordia, and a difficulty of breathing, at first perceived only in the act of ascending, or on using considerable exercise. The appetite decreases, the thirst augments, the urine is somewhat more scanty, commonly natural in colour, sometimes red, and when depositing a sediment (which is rare) this is commonly lateritious. There is an occasional cough, usually dry, or with slight mucous expectoration. In the progress of the disease, the countenance gets daily more pale, withered and anxious, but without œdema; the eyes are dull and heavy, and gradually lose their natural expression; and the lips pallid and thin. If the effusion is confined to one side, this is observed to become more rounded, and the intercostal spaces larger, as the water accumulates: This is followed by œdema of the same side of the chest, and sometimes of the arm of the same side,—a local affection unconnected with the œdema of the lower extremities, which afterwards comes on. At this period, percussion gives certain indications of the quantity of the fluid. During almost the whole course of this disease the patient can lie horizontally, on either side, or on the back; in the very last stage of it, he frequently lies obstinately on the affected side, if the effusion is partial, or keeps the sitting posture, if both cavities are full. There is never any palpitation of the heart, its action being observed to be feeble, tranquil, and regular. In accordance with this state of the heart, the pulse is often full, soft, slow and regular: as the disease approaches towards its final close, it becomes more feeble and frequent, but still remains regular. At this time the urine becomes scanty and thick. The disease retains a sameness of course, and the termination has nothing remarkable, being generally without much suffering, and with the senses and mental faculties still preserved." 53.

From this description of primary simple hydrothorax it is evident how illusory and false are many of those histories of this disease where, *starting from sleep—palpitation—præcor-*

*dial distress—inability to lie down—irregular intermitting pulse, &c.* are set down as pathognomonic symptoms—symptoms which clearly belong to an anterior organic disease of the heart or great arteries. Laennec also considers idiopathic hydrothorax as a comparatively rare disease—perhaps one in two thousand. This is a very small proportion. We never saw but one case, where no structural disease existed in any other organ. This was in a very robust young man, about 30 years of age, who laboured under cough without expectoration for some time, but did not emaciate, and continued going about his affairs up to the very day he died. He could lie perfectly horizontal, and even on both sides, but preferred lying on the right. He went to bed in his usual state, and was found quite dead in the morning. We had convinced ourselves by percussion that there was fluid in the right side; but had no idea of the extent of the effusion. Eleven pints of water, very little coloured, were taken from the right bag of the pleura—the lung of that side was not larger than a man's fist—the pleura itself was slightly opake and thickened—but we could find no structural disease in any part of the body. This was the first and the last case of idiopathic hydrothorax which we ever witnessed.

*Dropsy of the Pericardium.* Avenbrugger confounds the sero-purulent effusion into the pericardium, which is the effect of inflammation, with the colourless serous effusion, a morbid accumulation of the natural fluid. Corvisart, his commentator, properly restricts the term dropsy to the latter affection. This, like hydrothorax, is a very rare disease, as an *idiopathic affection.* It is generally accompanied, or rather preceded, by organic disease of the heart, lungs, or mediastinum. This being the case, we shall not advert to the diagnostic symptoms of *primary* hydropericardium—a malady too rare to afford diagnostic symptoms of sufficient accuracy to be depended on.

*Aneurism of the Heart.* With this disease, now so common, Avenbrugger appears to have been totally unacquainted —a circumstance that proves how diseases, and whole classes of disease, rise at one time, and disappear at another. A man like Avenbrugger, who so carefully studied diseases of the chest, would not have overlooked a class of organic diseases (now so common, and indeed so generally recognized, that every tyro considers himself capable of detecting them,) if they had then existed—at least in the proportion they now do. We have no doubt, indeed, that the prevalence of cardiac diseases has arisen out of the moral and physical circumstances of modern times, and that thus the evils to which flesh is heir will every day vary with revolving æras, per omnia secula seculorum.

Here ends the translation of Avenbrugger's work and Corvisart's commentary on it. Several able and important notes have also been added by Dr. Forbes, to whom the medical literature of this country is undoubtedly under obligations for having clothed the whole in an English dress, and thus rendered it easy of access to the whole profession of these isles and their various dependencies.

Next follows a brief outline of the principles of *stethoscopy*, (if we are permitted the expression) or method of distinguishing diseases of the chest by means of mediate auscultation, extracted from the great work of Laennec, and nearly as it stands in Dr. Forbes's translation, now out of print. We were about to pass over this portion (only 16 pages) entirely, as having been before the public in Dr. Forbes's former work, for some time past; but then we consider that this journal must necessarily pass through numerous hands where the original work has not been—and consequently that many of the cases (afterwards to be analysed) would lose much of their interest, from want of some knowledge of auscultation. On this account we shall dedicate two or three pages to a still briefer abstract of the principles of the stethoscope, which can be no great disadvantage to the adepts in the science, while to the noviciates it may be of considerable service.

Auscultation, or listening to the sounds produced in the interior of the chest by the natural motions of the organs of respiration and circulation, is not a modern discovery. Laennec has improved this diagnostic mean by the invention of the stethoscope or cylinder, which need not here be described. By practising with this instrument, we have no doubt that the sounds and movements from within are more distinctly conveyed to the ear, than when the naked ear is applied to the surface of the chest. But, as we before observed, simple auscultation is by no means a meagre substitute for mediate.

There are three kinds of exploration by the stethoscope;—that of the voice—the respiration—the circulation.

1. *Voice.* The action of speaking or singing, in health, excites a vibration through the whole parietes of the chest perceptible by the hand. This phenomenon ceases to exist where the lungs have become impermeable to air, or are removed from the parietes of the chest by the intervention of a fluid. But the utility of this sign is curtailed by a knowledge that numerous other causes modify or destroy it, as obesity, anasarca of the integuments, &c. The places to which the stethoscope is applied with most advantage, are, the axilla, the back, between the spine and scapula, and on the anterior and superior part of the chest, near the angle formed by the union of the clavicle with the sternum. When the stethoscope is applied to these points, the voice appears stronger and nearer to us than from the others. "If we apply the stethoscope to the larynx or trachea of a person in health, when speaking, we hear the voice of the individual as if coming directly from the point on which the instrument rests, and reaching our ear through the canal in it. In the second stage of phthisis, when tubercular excavations exist in the lungs, if the stethoscope be applied to the chest, over the site of one of these, the same transmission of voice through the tube is perceived. This phenomenon has been named *Pectoriloquism*: it is the pathognomonic sign of the morbid state just mentioned."

2. *Respiration.* The following cannot be condensed or abridged.

"On applying the cylinder, with its funnel-shaped cavity open, to the breast of a healthy person, we hear, during inspiration and expiration, a slight, but extremely distinct sound, answering to the entrance of the air into, and its expulsion from, the air cells of the lungs. This sound may be compared to that produced by a pair of bellows whose valve makes no noise, or, still better, to that emitted by a person in a deep and placid sleep, who makes now and then a profound inspiration.* We perceive this sound almost equally distinct in every part of the chest, but more particularly in those points where the lungs, in their dilatation, approach nearest to the thoracic parietes, as, for instance, the anterior-superior, the lateral, and the posterior-inferior regions.—The hollow of the axilla, and the space between the clavicle and superior edge of the trapezius muscle, exhibit the phenomenon in its greatest intensity. It is equally perceptible on the larynx, on the exposed or cervical portion of the trachea, and, in many persons, through the whole tract of this canal to the bottom of the sternum; but on the trachea, and in some degree at the root of the bronchia, the respiratory sound has a peculiar character, which evidently indicates the transmission of the air through a larger space than the air cells. In this position, also, it often seems as if the patient, in inspiring, inhales the air through the tube of the stethoscope, and expels it by the same, during expiration." 71.

The room in which auscultation takes place, should, of course, be perfectly noiseless. Contrary to what obtains in ex-

* "Its precise character will be best ascertained by applying the naked ear to the chest of a child." *Trans.*

ploring the voice, the respiration is heard even where there is excessive fatness, or anasarca. The sound is more distinct, in proportion as the respiration is more frequent. In children respiration is far more sonorous than in adults.

"When we can distinctly perceive, and with a uniform intensity, the respiratory murmur in every part of the chest, we may be assured that there exists neither effusion into the cavity of the pleura, nor any species of engorgement in the substance of the lungs. On the other hand, when we find the respiration is not to be distinguished in any particular point, we may safely conclude that the corresponding portion of the lungs within, is become impermeable to the air from some cause or other. This sign is as easy to be perceived as the presence or absence of the sound, in the percussion of Avenbrugger, and affords precisely the same indications. With the exception of some peculiar cases, in which the simultaneous employment of the two different methods gives us signs which are completely pathognomonic,—we may state it as a general fact, that the absence of the sound on percussion coincides uniformly with the absence of respiration, as ascertained by the stethoscope. Accordingly, this failure of respiration, existing in a greater or less degree, and over a greater or less extent of surface, is found to be a principal distinctive sign of Peripneumony, Pleurisy, Hydrothorax, and all other diseases, which in any way obstruct or impede the natural action of the lungs." 74.

There are several modifications of the natural respiratory sound, in consequence of its combination with other sounds in certain morbid states of the lungs, but these must be studied by the auscultator after he has made some progress in the art.

3. *The Circulation.* This is studied in relation to sound, impulse, and rythm.

"In ordinary circumstances, the stethoscope, applied between the cartilages of the fifth and sixth ribs, at the end of the sternum, &c. conveys to the ear a distinct sound, even where the pulse is very feeble, or imperceptible. This, in the healthy body, is double, and each beat of the arterial pulse corresponds to this double sound, in other words, to two sounds. One of these is clear and rapid, and somewhat resembles the sound produced by the valve of a pair of bellows: this corresponds to the systole of the auricles. The other is more dull and prolonged, coinciding with the beat of the pulse and with the shock or impulse communicated to the parietes of the chest by the motion of the heart:—it indicates the contraction of the ventricles. The sounds heard at the end of the sternum are produced by the action of the right side of the heart; those between the cartilages of the ribs by the left cavities. In the state of health the sound produced by the contractions of each side is the same." 76.

This sound is great in proportion as the parietes of the ventricles are thin and their impulse feeble—consequently it does not arise from percussion of this organ against the side. In a moderate degree of hypertrophy, or thickening of the heart's substance, the ventricular contractions produce only a dull sound, like the murmur of respiration :—in a high degree of hypertrophy, the contraction of the ventricles yields merely a shock without any sound. On the other hand, when the ventricular parietes are thin, the noise produced by their contraction is clear and loud, approaching to that of the auricles. In health and good proportion, the sound of the heart is only heard in the cardiac region—in thin persons and children, however, it is more extended. When the cardiac pulsations are heard beyond the region in question, the individual is seldom in good health. If he have no formal dyspnœa, he has, at least, shorter respiration than natural, is easily put out of breath, and is subject to palpitation. This state, however, which is that of many asthmatics, may remain stationary many years, and does not always prevent the attainment of old age. It may be taken as a general fact, that the extent of sound is in the direct ratio of the thinness and weakness of the heart, and, consequently, inversely as its thickness and strength.

*Impulse.* The degree of this is, in general, inversely as the extent of *pulsation*, and directly as the thickness of the walls of the ventricles.

"In a person whose organs of circulation are well proportioned, this impulse is very little perceptible, often quite imperceptible, especially if the individual is rather fat. When the parietes of the heart are unnaturally thick, the impulse is usually so great as very sensibly to elevate the head of the observer, and sometimes to give a disagreeable shock to the ear. The more intense the hypertrophia, the longer time the impulse is perceptible. When the disease exists in a high degree, we feel as if the heart, in dilating, first comes in contact with the thoracic parietes in one point only, and then with its whole surface, and that it contracts and falls back all at once. The impulse of the heart is only felt during the systole of the ventricles; or if, in some rare cases, an analogous phenomenon accompanies the contraction of the auricles, this is easily distinguished from the former. In fact, when the systole of the auricles is attended by any sensible impulse, this is perceived to have its seat much deeper; and most commonly it consists merely of a sort of vibration. In any case, it is very little marked as compared with the sensation produced by the contraction of the ventricles, when these are of a good degree of thickness.

"When the parietes of the heart are thinner than usual, no impulse is communicated, even when the sound is the greatest; and, in this case, the alternate contraction of its cavities is only distinguished by the sound these produce. A strong impulse, therefore, must be regarded as the chief sign of hypertrophia; and the absence of all impulse as the characteristic of dilatation of the heart." 80.

The subject of the rythm of the heart's action, or the ratio between the action of the different chambers of that organ, must be an ulterior study, and need not detain us here.

This slight sketch may seem unnecessary to the few who have studied the subject; but, as not one in ten of our readers are auscultators, the little we have laid before them may possibly stimulate to further enquiry, in which case, our object is fulfilled.

We now come to the cases and dissections furnished by Dr. Forbes himself, being thirty-nine in number, and occupying nearly 200 closely printed pages. They are principally dispensary cases, and consequently of the most authentic class. This part of the volume indeed is extremely valuable, and is highly creditable to the zeal, the talent, and the candour of the author. If Dr. Forbes persists in the laudable and industrious course which he is now pursuing, he will not only be the architect of fame and fortune for himself, but prove a benefactor to humanity and an honour to the profession. We can select but a very few of these interesting cases for specimens or illustrations in this article—but we hope the work itself will circulate wherever the English language prevails. We shall confine ourselves principally to cases where dissection proved the truth or error of diagnosis and prognosis.

*Case* 1.—G. R. a black-smith, aged 30 years, had been ill about five months, the principal symptoms being pain in the præcordia, indigestion, dyspnœa, palpitation, inability to lie in the horizontal posture, disturbed sleep—and latterly, cough with mucous expectoration. The pulse is extremely full, strong, and vibrating—the action of the heart great, and occasions an obvious tremor of the head. Examined by the stethoscope, though slightly and imperfectly, the diagnosis was hypertrophy of the heart with enlargement of the ventricles. "The action of the heart perceptible over the whole anterior part of the chest: the *impulse* very great, and the sound very loud and peculiar." Bleeding gave great relief, with mercurial purgatives, and digitalis: but the cardiac symptoms made progress. About three months afterwards, he was again examined by the stethoscope, and this examination we shall give in the author's own words.

"August 11th and 15th. Examined with the Stethoscope more particularly. *Impulse* of the heart's action is very great in the region of the heart, and is very perceptible, though in a less degree, to a considerable distance beyond, viz. under the right clavicle, and over the greater part of the anterior of the chest: the *sound* of the heart's action is very loud in the region of the heart, and is very distinctly perceptible over the whole chest, before and behind, except on the lower part of the right side; it is, however, much louder over part of the right side, especially under the clavicle and along the whole extent of the sternum, than either in the region of the heart, or over the left side. The sound is much more perceptible when the stopper of the instrument is removed; it is thus heard to be extremely loud under the right clavicle, and at the top of the sternum, and resembling the grating noise of a saw with fine teeth, or that produced by the forcible action of the piston in a large empty syringe.* It is much louder under the right clavicle than the left; and it is also heard very distinctly *above* the clavicles. In the action of the heart, as studied by the stethoscope, the sonorous contraction of the ventricle seems to occupy by far the greatest part of the whole space of time,—the contraction of the auricles, and the subsequent interval of repose, being extremely brief. The impulse or shock of the heart's action is not synchronous with the pulse of the radial arteries. I am not sure that there is either swelling or pulsation of the jugulars; but there is a very perceptible tremulous motion of the whole throat, connected with the pulsatile tremor of the head formerly noticed. Applied over the carotids, the stethoscope conveys a very loud sound, especially on the right side, on which the pulsation is much stronger, and accompanied by a jarring vibratory sensation scarcely perceptible on the other. The whole thorax in the region of the heart is very painful on pressure.

"The sound of *respiration* is very indistinct over the whole chest, being drowned, probably, by the very loud sound of the heart. *Percussion* elicits a very dull sound in the region of the heart, and to some distance around; but over the rest of the chest, the sound is natural. Pulse 100, regular, full and strong, but less strong than formerly.

"The swelling in the præcordia is equable, and conveys the idea of a large solid body, like the liver, being suspended there, and hanging down nearly as low as the umbilicus: it seems to extend as far on the left side as the right, and is painful on pressure.

"DIAGNOSIS. *Great dilatation of the right ventricle with slight hypertrophia; and moderate dilatation of the left ventricle with very considerable hypertrophia: In other words:—Dilatation of both ventricles,—greatest of the right; Hypertrophia of both ventricles,—greatest of the left. Dilatation of the Aorta? Great enlargement of the left lobe of the liver.*" 87.

"* I noted down these comparisons at the time (as well as every other statement in the case), but in summing up my diagnosis, it will be seen that I neglected to draw from them some very obvious conclusions, respecting the state of the valves of the heart."

The prognosis was, of course, DEATH. This event took place two months afterwards, preceded by that dreadful state of suffering, which characterises this most melancholy class of human afflictions.

"Totally incapable of bearing the horizontal posture, he remained, day and night, seated, generally in his chair, sometimes on his bed; with the constant sense of the most distressing uneasiness, and occasionally of the most acute pain, in the region of the heart,—almost entirely deprived of sleep, not daring to move for fear of inducing a paroxysm of pain and palpitation,—and anasarcous over the greater part of the body. His only relief was from blood-letting; and this was very frequently repeated. He took a variety of medicines, partly in hopes of relieving the original disease of the heart and liver, partly with the intention of removing some of the consequences of this,—the anasarca, the pain, and sleeplessness; but with very little benefit, except from blood-letting." 88.

*Dissection.*—Present, Mr. W. Guy, Dr. Conolly, Mr. Tully, and Dr. Forbes. The note of the diagnosis was read to these gentlemen, before the body was opened. Pericardium sound, and very little fluid in its cavity. The heart itself enormously enlarged. The parietes of the *right* ventricle were from three eighths of an inch to half an inch in thickness, and uniformly so. The cavity of the ventricle was capable of containing a large-sized lemon. The *right* auricle was enlarged in proportion. The cavity of the *left* ventricle was smaller than that of the right by nearly one third—and the parietes an inch in thickness. The mitral valve was sound. The aorta was hard, and somewhat contracted at its origin; but beyond that, considerably dilated as high as the arteria innominata. The semilunar valves of the aorta were much thickened, hard, and rough. These and the interior of the vessel were encrusted with osseous deposits. The pulmonary artery and valves escaped notice. The whole substance of the lungs, especially on the right side, exhibited the characteristic induration of peripneumony in a slight degree. The inferior lobe on the right side was *hepatized*. The liver was greatly enlarged, particularly the left lobe. Its substance was harder than natural, and of a mottled appearance.

*Case* 2.—A hard working labourer applied to the dispensary on the 8th of April, 1823. He had been seized rather suddenly three months previously with oppression and tightness across the chest, dyspnœa, &c. accompanied by irregular pulse. He has not been able to work since. It comes on in paroxysms, but is never entirely absent. The legs are now œdematous.

"STETHOSCOPE. *Impulse* of the heart, in the cardiac region, very great, sound also louder than natural in the same place, and audible over the whole chest, before and behind, and especially along the *right* side of the sternum. On the back, the sound (owing to the frequency of the heart's contraction) is rather that of a continuous murmur, than of distinct pulsations. With the exception of irregularity of the rythm, there is no other peculiarity in the sound. *Respiration* natural. To this note of examination, I added the following DIAGNOSIS and PROGNOSIS: *Dilatation with Hypertrophia of the heart; dilatation especially of the right cavities. Death.*" 97.

Dr. Forbes directed the loss of some blood, and some aperient medicine. In about a fortnight the man's daughter came for his discharge, informing our author that her father had been at work ever since! This information was not a little startling to Dr. Forbes, who began to look over his notes, somewhat mistrusting the fidelity of "son cylindre" and its diagnosis, as well as his own prognosis. But a few more days proved the correctness of both. The man was suddenly seized with one of the paroxysms, while sawing a piece of wood in his garden, and in twenty-four hours expired. Previously to examination, and in the presence of several medical gentlemen, Dr. Forbes voluntarily committed himself and the credit of the instrument by the following written statement of his expectations:—"*Whole heart enlarged—principal dilatation of the right ventricle. No contraction of valvular orifices. Lungs sound.*"

*Dissection.*—Present, Mr. Guy, Mr. Dods, Dr. Forbes. All the viscera of the thorax and abdomen, except the heart, particularly sound. The heart itself was about two-thirds larger than natural, the greatest dilatation being in the right ventricle and auricle. There were no marks of inflammation—no valvular disease.

This case shews the importance of auscultation, and affords an example of the delusive gleams of hope which sometimes break in upon diseases of the heart, only to render the subsequent disappointment more poignant.

Dr. Forbes makes some judicious observations at the close of the above case, one of which we shall quote, as highly deserving the attention of the young.

"As bearing directly upon the present subject, I would here beg to suggest a most important caution in the use of the Stethoscope in diseases, or supposed diseases, of the heart. It is—never to overlook the precept of Laennec, not to explore the circulation, unless during the most perfect state of quietude, both bodily and mental, of the patient. "*We must never deduce any conclusions from the analysis of the heart's pulsations, unless this has been made after a sufficiently long repose, if the patient has been taking exercise; or during the most perfect calm and quietude, if he already labours under disease of the heart.*" (Tom. ii. p. 228). I am the more anxious to impress this caution on the mind of the reader, as it is of especial importance in Dispensary practice, where patients generally walk to the place of examination, and where trials with the Stethoscope are more likely to be made than in private practice. I am conscious of having, more than once, committed serious mistakes, from inattention to this circumstance: and I shall, in the present volume, record one or two instances of the kind, with the view of impressing it more deeply on the attention of the young Auscultator." 101.

*Case* 3.—A man, 40 years of age, a German by birth, was admitted a patient in the Chichester Infirmary, under Dr. Conolly, in February, 1823. He had long been suffering from some disease of the chest, attended with such a violent cough as to be an annoyance to the neighbourhood. There was also purulent expectoration. Dr. F. first visited him in March, 1823, at which time he was confined to bed, and in a great measure incapable of swallowing from some obstruction. Examined with the stethoscope on the 23d March, the following note was made.

"*Action* of the heart regular; *impulse* considerable in the region of the heart; *sound* louder than natural in the same place and as low down as the angle of the short ribs, also towards the sternum; most audible with the open stethoscope; both the sound and *impulse* perceptible at the very extremity of the xiphoid; *sound* perceptible over all the right side (anteriorly) but not very loud,—more so in the middle than under the clavicle; about the nipple (right) and below it, there seems to be *impulse* as well as sound. *Respiration* audible over the grearer part of the *left* side (anteriorly); attended, in the vicinity of the heart and under the clavicle, with a dry sonorous rattle, very loud; *puerile* under the clavicle, and extremely puerile and without any rattle, under the sternal extremities of the 2nd and 3rd ribs; very indistinct over all the right side, yet still in some degree perceptible in most places, and, where perceptible, accompanied by a slight crepitous rattle. Pectoriloquism not explored, except under the left clavicle, where it is wanting." 134

This was three weeks previously to the patient's death, and upon looking over it before opening the body, the following diagnosis was committed to paper, and shewn to the medical gentlemen who assisted in the dissection.

"STEINICKE. *Dilatation with Hypertrophia (slight), especially of the right ventricle: heart otherwise natural? Left lung sound, especially in the superior lobe. Inflammation and consequent consolidation (in a considerable degree) of the greater part of the right lung. Larynx or Trachea diseased, and affecting the œsophagus?*"

*Dissection.*—*Right lung* adherent in every point to the parietes of the cavity—its substance hepatized and tuberculated. In the posterior part of the upper lobe there was a vast hollow abscess capable of containing an orange. The *left* lung was partially adherent, but was crepitous, with partial indurations and unsoftened tubercles. The *heart* was of natural size, but adherent to the pericardium in every point, without any obvious intermediate tissue. The right ventricle and auricle were sound; and so were the left, except some ossified columnæ carneæ on this side. A large tumour consisting of a congeries of indurated glands, was found under the upper part of the sternum in the posterior mediastinum, between the aorta and bifurcation of the trachea, compressing the œsophagus. The liver was sound, but descended lower than usual. Other viscera natural.

It will be perceived that the diagnosis respecting the lungs was perfectly correct, while that which regarded the heart was erroneous. This error, however, is pardonable, when we come to consider the adhesion of the heart to the pericardium, which gave to its action that impulsion which led our author to conclude that hypertrophy had taken place. There is, in fact, no certain diagnostic mark laid down even by Laennec, for pericardiac adhesion. In all the instances which we have seen there was violent action of the heart—but this is common to many other affections of the same organ.

*Case* 4.— Mr. N——, aged 40, had all his life been subject to constipation, heart-burn, head-ache, and other symptoms of indigestion; but was nevertheless strong and hearty till within two years of the above age. He then began to have occasional fits of breathlessness and palpitation, especially on ascending any eminence, which symptom progressively increased till the time of consultation, December 9th, 1822. There is sense of fulness and of oppression in the region of the heart. Since the thoracic symptoms have appeared, he has, in a great measure, lost his head-ache and heart-burn, but the bowels continue obstinately costive, and he has some nausea and retching. He has also become subject to very severe cough, mostly dry, but latterly with mucous expectoration, tinged at times with blood. He can take a deep inspiration without difficulty, and has no pain in any part of the chest, except during the paroxysms of great dyspnœa accompanied by palpitation. He has been occasionally jaundiced. His urine is now scanty, high-colored, and lateritious. He has been unfortunate in business and suffered great mental distress. He is now very low-spirited. On examination, a swelling and hardness, answering to the left lobe of the liver, were observable in the præcordia. On percussion, the chest sounded well in every part, except in the region of the heart, where the sound is very dull over a considerable space. Not examined with the stethoscope. Bleeding to 16 ounces gave great and immediate relief. The pulse is above 100, small and regular. Tongue pretty clear—stools very dark—sickness and retching distressing. Topical bleedings from the præcordia—blistering—mercurial frictions—gentle tonic aperients and alteratives. From this treatment he derived great relief. The sickness and retching vanished, and the other symptoms were much ameliorated. A few days after the first examination the stethoscope was applied, with the following results: the respiratory sound is quite wanting on the *left side* from below the 3d or 4th rib, to the bottom of the thorax, over a space bounded by the rib abovementioned, the sternum on the right, and a line drawn about an inch outside the nipple on the left—in other words, over a space as much as a spread hand could cover, the heart being the centre. Over the remainder of this side the respiration was indistinct. Over the *right* side of the chest it was very distinct. The sound of the heart's contractions was very loud over all the space where the respiration was indistinct. It is also distinct, though not loud, over the rest of the left side. From these and other phenomena, Dr. Forbes ventured to prognosticate, "*considerable dilatation of the heart, with moderate hypertrophia.*" The lungs he considered as sound, and no hydrothorax at that time.

7 *January*. The symptoms worse—more dyspnœa—anasarca of the limbs—considerable impulsion of the heart—pulse 120, and small. Infusion of digitalis brought on a plentiful diuresis, with diminution of the external swellings, and great relief of the dyspnœa and uneasy feelings in the chest. But the relief, as usual, was only temporary, and superseded by a new evil—extensive erysipelas of the right leg, terminating in sphacelus. Stimulants were now, of course, administered—the pectoral symptoms were relieved—the sphacelation was arrested—and the slough separated. Cicatrization had nearly been effected, when the pectoral symptoms returned in an aggravated degree, and continued to progressively increase in violence to the fatal close. In this melancholy progress the stethoscope detected hepatization of the superior lobe of the right lung, in addition to hypertrophy of the heart. He died on the 14th April, after a long series of dreadful sufferings.

*Dissection.*—Liver very large—of a dark mottled colour externally, somewhat rough, but not otherwise diseased. Its substance was more solid than natural, and of a pale colour. It was much gorged with blood—many gall-stones in the gall-bladder. On removing the sternum and cartilaginous ends of the ribs, the whole space exposed was found to be occupied by the pericardium (no part of the lungs being visible) distended with fluid, of a sero-purulent character, and upwards of a *quart in quantity!* The whole internal surface of the pericardium was covered with a layer of white curd-like substance, of a frosted appearance, and the product, unquestionably, of inflammation. Here were seen those sacculated compartments resembling the stomach of the calf, produced by the crossing of bands of the adventitious membrane from side to side of the distended sac. On the left side of the heart there was a mass of tremulous jelly, hanging in folds, half an inch thick in some places. The membrane underneath was very red, not uniformly, but in patches. The heart itself was probably one half larger than natural. The parietes were not increased or decreased in thickness. The valves were all sound. The *right* lung was adherent to the ribs in different points—much compressed and displaced by the distended pericardium, but still crepitous. The lower part of the *left* lung was compressed and hepatized. In this side of the chest there was about a quart of sero-purulent fluid, of the same kind as that in the pericardium. For Dr. Forbes's remarks on the above case, we must refer to the volume itself.

*Case* 5.—The following case cannot be abridged without injury, and is moreover very short. We give it as a specimen of functional, imitating organic disease—especially in females. Few days pass that we do not see shades, lighter or deeper, of the same—and great error committed in the prognosis as well as treatment.

"*Sympathetic Angina Pectoris.*"

"*August* 12, 1822. Miss ———, ætat. 43. This patient, a lady of a cultivated intellect and sensitive temperament, describes herself as having been always a bilious subject, yet pretty strong and active, and in the habit of taking occasionally great bodily exercise. About two years ago she began to be affected with dyspnœa on going up stairs. This affection gradually increased, and, after a short time, was excited by the slightest motion. In the course of a few months, in addition to the dyspnœa, a violent paroxysm of pain under the sternum, shooting to the back and down both arms,—in short a paroxysm of *angina pectoris*,—was produced by almost every attempt at motion. This state of things has continued to the present time, or rather seems to have been progressively getting worse. *Now*, she cannot walk across the room, or dress herself, or indeed use any kind of bodily movement, without bringing on the paroxysm. She is also subject to frequent palpitation, even, when at rest, and to a constant and most distressing noise in the ears. But for these two last named symptoms she feels tolerably well, when lying still. She has no permanent dyspnœa whatever, nor pain nor stricture of the chest, and never appears to have been affected with any acute inflammation of any of the thoracic viscera. She is never hot or feverish, although her skin, she says, is always dry. She can lie indifferently on either side or on the back, and with the head low, and her sleep is undisturbed by frightful dreams or sudden startings, but broken, after intervals of a few hours, by an attack of palpitation.— There is no indigestion properly so called, but the bowels are torpid, and the appetite impaired. The catamenia are regular. She has been for four years subject to a considerable discharge from the hæmorrhoidal veins, coming on regularly after every menstrual lustration: this discharge has not been present for six weeks. Pulse 90, quite regular and of moderate strength. Tongue clean. Urine of natural quantity.

"I applied the stethoscope twice in this case. At my first visit, the sound and impulse of the heart were found moderate in the region of the heart, but the former audible under the right clavicle. [At this time the patient was somewhat agitated]. At my second visit, on the 23rd, she having remained perfectly still and in the recumbent posture, by my recommendation, the action of the heart was found perfectly natural, both in rythm, impulse, and extent of pulsation. The respiration also was found to be natural.

"Considering this case as one in which the affection of the heart was most probably nervous or sympathetic, or in which the organic derangment, if any, was but slight,—after ordering a few oz. of blood to be taken away as an experiment (and the loss of which was not beneficial), I enjoined absolute rest in bed, and prescribed the following mild alterative pill and draught, with the view of improving the digestive powers and the intestinal functions:—Pil. Hyd. Ext. Col. c Ext. Hyoscyami aa gr. j. Ipec: Pulv: gr. $\frac{1}{3}$ M. ft. pil. ter quotidie sumenda. Haust. merid. ex Infus. Rhei, Infus: Calumbæ aa dr. vj, Spirit. Myrist. dr. ss.

"This plan seemed to be productive of great benefit, as the appetite and strength improved, and the paroxysms gradually disappeared under its continued use. It was necessary, however, for the patient to confine herself for many months. She has been now (Nov. 1823) for more than half a year comparatively well.

"May 1824. She still remains comparatively well, occasionally subject to slight palpitation, but never to any of the severe paroxysms of angina." 177.

We agree with Dr. Forbes in considering the above case as an example of "the first stage of a numerous class of organic diseases of the heart, namely, disordered action of the heart, arising in bilious and nervous subjects, and proceeding, if unchecked, to irremediable disorganization." We are quite certain, however, from repeated observation, that this "first stage," if it can strictly be called so, will often go on for years, without any material change of structure—at least irremediable change, and ultimately disappear altogether. Still this disorder of function occasionally runs quickly into alteration of structure, and therefore should never be treated lightly, or neglected.

In these cases, the examinations by the stethoscope, percussion, &c. are very useful, as furnishing *negative* results, and satisfying the practitioner of the absence of organic disease.

We have said that Dr. Forbes was a candid as well as a talented physician. There is indeed a tone of ingenuous candour pervading the whole work which charms—and must ever charm. We shall offer a proof.

*Case* 6.—James Waller, ætat. 23, applied for dispensary assistance on the 23d of May, 1823. He had been very strong and healthy (being a day-labourer) until the preceding October, at which time he fell, with a heavy sack of wheat on his back, pitching with his *left* breast on the sill of a door, with a shock of extreme violence, followed by extreme pain in the chest, and instantaneous discharge of blood from the lungs, which continued for some days. The pain went off in a few days, but was followed by dyspnœa and swellings of the legs.

"This dyspnœa was at first severe, and, for several months, constant, whether quiet or not; latterly it has been less, and little urgent or indeed perceptible except on motion. He continued at work for two months after the accident, but was then obliged to desist on account of a return of the spitting of blood and a severe pain in his *right* side. This pain went off after about a month's continuance, and he was thinking of returning to his labour, when he was suddenly seized with a most violent pain in the region of the heart, attended by excessive palpitations. This pain lasted, also, about a month, and then went off; but the palpitations have never subsided. During the first fortnight after the sudden attack of pain in the region of the heart, he was altogether unable to lie down; but previously to that period, and since, and *now*, he can lie down with his head as low as when in health, and with equal facility on either side; and he sleeps very soundly, without dreaming or alarm. Ever since this attack in the left side, *stooping* produces vertigo, and a state which he compares to the effect of intoxicating liquors. All kinds of bodily exertion, such as walking, and especially going up hill, increase the palpitation or habitual state of over-action of the heart, and augment the feelings of distress constantly referable to that spot. His appetite and bowels are regular. His mind is evidently very considerably depressed, and he says his thoughts are confused.

"The pulsation of the heart is *seen* very distinctly over the left side of the chest; the impulse is felt by the hand to be very great, and very perceptible across the lower half of the sternum as far as the right nipple. The carotids are also seen and felt beating very strongly all along the neck. The pulse at the wrist is not very full, but very strong and regular, about 100.

"STETHOSCOPE. *Impulse* of the heart is extremely great in the cardiac region, so as to convey a strong shock to the head, through the instrument; but there is very *little sound*. The impulse is synchronous with the pulse; and there is scarcely any shock or sound to be perceived answering to the contraction of the auricles. The impulse or shock, without sound, is strongest in the line of the nipple, and four or five inches below it. Under the middle of the left clavicle there is a very distinct and loud sound (with very slight impulse) resembling a hollow murmur, and almost *single*;—that is, there is only one action or sound, answering to the contraction of the ventricles (the pulse). Over the left axillary face, the sound is very loud, and is not so much a grating intermitting noise, as a regular, continuous, hollow tone; a similar sound is very distinct a little way to the left of the nipple, on this side. Sound and impulse very distinct over the lower half of the sternum; and the *sound* very loud over all the right side anteriorly, and without any grating or roughness; perceptible, also, over all the back, on both sides, but much less loudly than anteriorly.

"*Respiration* not much explored,—audible on the back, but drowned before by the loudness of the sound of the heart's contractions. *Percussion* does not give pain in the region of the heart, but does under the right nipple, over the site of the pain formerly experienced there. He is constantly sensible of a great and very disagreeable sound in his ears: which I found, on trial, to be greatly and instantly relieved by compression of the carotids. The præcordia and right Hypochondriac region appear to be very full.

"His complexion is still very fresh and pinky; but he says he is much paler than when in health.

"Diagnosis. Hypertrophia with Dilatation of the Heart—in a great degree, without valvular disease. Hypertrophia principally of the left ventricle? Hypertrophia with dilatation of the right? Prognosis.—Death." 189.

On the 2d of January, 1824, or seven months after the fatal sentence was pronounced, Dr. Forbes was somewhat astonished to meet his patient, who informed him, he had been following his labours, as a man servant, for the last five months—having returned to work about two months after Dr. F. saw him!—He was, of course, greatly amended, though not quite well. Hard labour still excited palpitation and dyspnœa. The rhythm of the heart was now regular, and the impulse not much above par. The sound, however, was still loud under the sternum, and over the right side. Upon being questioned, the man now admitted that he had been under a considerable degree of mental agitation during the former examination, although he denied it at *that* time. He had also walked to the Dispensary, which had increased the action of the heart. This case offers an instructive example of the errors which may be committed by forming a diagnosis or prognosis in cardiac diseases from a single examination, and that at a time when the function of the organ is disturbed by any mental emotion. Dr. Forbes candidly confesses that he mistook a transient excitation for an habitual condition, and was thus led into an erroneous, or at all events, an exaggerated diagnosis and prognosis. Still he is inclined to think (we hope he is mistaken) that the error was not in *kind* but only in *degree*. Time alone can solve this point, and Dr. F. promises to disclose the result to the public, whatever way it turns out.

*Case 7. June 3d*, 1823.—John Parsons, ætat. 45, a farmer's servant, has been affected about a month with various ailments of which he can give but a very imperfect account. He seems to have much dyspnœa, and has a very anxious countenance; complains of pain in the præcordia, where there is tenderness on pressure. Urine high-coloured—tongue furred—pulse 80 to 90. He was bled, and had aperients with diuretics. He received little or no benefit from the treatment adopted, and and on the 19th of July, Dr. F. examined him with the stethoscope. The sound of the heart was natural, with little or no impulse. Over the whole of the right side of the chest the respiratory sound was almost entirely inaudible. Percussion is in accordance with the stethoscope, and elicits only a very dull sound, while on the opposite side the sound is clear. When first taken ill he could lie on his back—after a time he rested chiefly on the *right* side—now, he can lie equally well on either side, on the back, and also with the head low. He does not start from sleep. His urine is high-coloured, but of natural quantity. DIAGNOSIS. "*Chronic pleurisy, with copious effusion, in the right side.*"

Between this and the 27th October, he rather mended than otherwise. It was then discovered that *contraction of the right side of the chest* had taken place to a very considerable extent, the whole trunk being swayed to that side, and the left shoulder being much hgher than the right.

In the trials of percussion and mediate auscultation, the contracted side still sounds perfectly dull. *November 25th.* Has continued slowly to improve; but complains of slight pain in the lower part of the right side. His breath is pretty good, and he has no cough. *December 19th.* Still better—gains flesh and strength. The contraction of the right side is increased. Respiration is now heard in the upper part of the affected side, but not below the line of the nipple. *April 2nd.* Has had pain, with dyspnœa. Bled and blistered. 7th. Great dyspnœa—expectoration of thick mucus mixed with blood—pulse 120, small and feeble—urine very scanty. *Diagnosis.* "Supervention of acute peripneumony on both sides." Venesection—blisters—antimony &c. He died on the 10th, but unfortunately permission was not obtained to examine the body. There can be no doubt, however, of the existence, in this case, of chronic pleurisy, with effusion, partial absorption, and contraction of the chest on that side. It is probable, too, that the case would have ended favourably, as some of Laennec's did, had it not been for the accidental supervention of acute inflammation, of which, and not of the original disease, he evidently died. Another case of contraction of the chest, with chronic pleurisy, is next related by Dr. Forbes, and the event was still doubtful when his work went to press. From these and other cases which he has seen, he is led to be surprised at Mr. Shaw's scepticism respecting Laennec's statements on this point of morbid anatomy.

*Case* 8. *January* 22*nd*, 1824. Mrs. E——, ætat. 53, has been subject, for several winters past, to some cough, but was still able to attend to her affairs until six months ago, when she was attacked with inflammation of the chest that required bleeding repeatedly. Since that period she has been ailing, and during the last two months she has been much worse. She has paroxysms of violent cough, with scanty expectoration of white viscid mucus, occasionally tinged with blood—no pain of chest, but a slight pain about the larynx on taking a deep breath. There is dyspnœa when any bodily exertion is used —pulse nearly natural, as are the bowels and appetite. She cannot lie on the *right side*, and, on examination, this side of the chest, under the clavicle, seems more compressed than the other.

"On applying *Percussion*, the difference of sound on the two sides was so very great, as to be, at once, spontaneously remarked by the patient herself, and the by-standers, at the time;—the sound of the left side being very clear, and that of the right quite dull,—indeed *fleshy*. The *Stethoscope* gives analogous results,—the respiration on the *left* side being very loud, indeed puerile, and only just barely perceptible on the right. There is no *rattle*. *Hægophonism* appeared to me distinct on the right side;—at least there is a kind of *punchinello-note* very audible on this side and not at all on the other.

"Diagnosis. *Chronic Pleurisy, with copious effusion, of the right side*." 248.

A strict vegetable diet was recommended—leeches and a blister to the right side—an aperient emulsion.

*March* 8*th*. She has lost flesh—the pulse is up to 110—can still lie only on the left side. Same results from the stethoscope and percussion. *Prognosis*. "Death within the year." *May* 11*th*. Appears to be gradually losing ground. She has returned to animal diet and dismissed her medical attendant. *June* 24*th*. Considerably worse—has lost much flesh and strength. She remains, for the most part, sitting in bed, as any attempt to assume the horizontal posture or walk, aggravates the dyspnœa and cough. Sleeps badly, and awakes frightened. The cough is severe in paroxysms, and the expectoration still pure frothy mucus, occasionally tinged with blood. The dyspnœa is constant—no pain in the chest, but general uneasiness in her limbs—she is pallid—skin cool—perspires copiously at times, especially about the chest—pulse 108, and weak—appetite bad—tongue, bowels, urine natural—legs slightly œdematous. Same results as formerly with the stethoscope and percussion. *July* 4*th*. Died this morning, the symptoms having progressively increased.

*Dissection*—in presence of Mess. Williams, Tully, Hills, and the Author. Percussion of the chest still elicited the dull sound on the *right*, and natural sound on the *left* side. A scalpel being pushed into the right side of the chest, a stream of dark yellow serum issued forth. The right side was completely filled with the same kind of fluid, three pints in quantity—the right lung was compressed on all sides towards its root in the mediastinum, into a compact rounded body, like a couple of closed fists. Its substance was harder than sound liver, and closely resembles what is termed scirrhous liver. It sunk in water. The whole of the pleura on this side had lost its natural character, being white, and without the glossy lustre of a serous membrane. It was also rough and uneven to the touch, apparently owing to a sort of fibrous, or cartilaginous degeneration. The membrane was, upon the whole, three or four times its natural thickness. There was no mark of *recent* inflammation. The *left* side contained about half a pint of serum, but the lung and pleura were nearly sound. The heart was flaccid, but of natural size. No other morbid appearance.

Dr Forbes thinks (and we quite agree with him) that, in the above case, the disease of the pleura (whatever was its nature) and the consequent effusion, were the primary affections—the compression and hepatization of the lung being secondary. The correctness of the diagnosis furnished by auscultation and percussion is sufficiently obvious.

Here Dr. Forbes makes some observations on the subject of removing the fluid from the chest by means of a surgical operation, in cases of hydrothorax and chronic pleurisy. He had before remarked that the adhesions which exist in many cases of chronic pleurisy would render the operation nugatory, if not dangerous, and so would disease to any extent, in the lungs.

"When, however, the accumulation of fluid is copious, and the disease of not very long standing, and if there is good reason for believing the lungs not to be primarily affected, I presume that the operation is not only justifiable, but even very advisable, in many cases." 257.

This operation is of very ancient date, as may be seen in Sprengel's History of Medicine (" *De l'Operation de l'Empyème,*" *vol.* ix. *p.* 1 *to* 90.) and has been practised or recommended by numerous writers, from the time of Hippocrates downwards, including Avenbrugger himself. The disuse of paracentesis thoracis, in this country, is thought by our author to result, in part at least, from overlooking chronic pleurisy—the usual form of empyema. From this, he does not mean to advocate the practice generally, but only thinks it would be well not to lose sight of it altogether, since it is a measure which has been reported as often successful, and that too in a disease which is generally, if not always, beyond the power of medicine. The following are Laennec's sentiments on this point.

"With regard to the operation itself, I am of opinion that it is one of much less severity than is usually imagined. Its success depends less on the condition of the pleura, than on that of the lungs; and when this viscus is not too deeply affected by numerous tubercles, or by a large gangrenous eschar, it ought almost always to succeed. The admission of air into the cavity of the pleura is probably, also, less dangerous than is commonly believed. This is, indeed, proved by the cases of wounds of the thorax, and the history of recoveries after the operation of empyema.

"I have not met with any example of acute inflammation of the pleura supervening to the operation; and I am not even sure whether the supervention of such an inflammation might not be the means of a speedy and certain cure of the disease, by producing an union between the lungs and ribs. At all events, in cases where the severity of the symptoms presented little hope of a cure from the operation, some benefit, and no danger, might result from simply puncturing the chest. Perhaps, even, it might be useful to draw off the fluids in this manner in all cases of chronic pleurisy, repeating, if necessary, the puncture, five or six times. This slight operation would be attended with no inconvenience, and the puncture will heal up immediately." 259.

We have now, we hope, presented our readers with sufficient samples of the work before us to enable them to form a tolerably good estimate of its merits and importance. We think indeed that few will peruse this article and fail to place the original work in their library, for reference and study. If they do not, they injure themselves more than they injure the author. Dr. Forbes has added an appendix, containing an admirable epitome of a little work by Dr. Collin, on exploration of the chest by percussion, auscultation &c. lately published in Paris. It is avowedly a compilation, and only contains what is to be found in the works of Corvisart, Avenbrugger, and Landré-Beauvais. It is not necessary, therefore, that we should take any farther notice of it in this place. We part from Dr. Forbes with sentiments of respectful esteem.

